# Understanding the Prooxidant Action of Plant Polyphenols in the Cellular Microenvironment of Malignant Cells: Role of Copper and Therapeutic Implications

**DOI:** 10.3389/fphar.2022.929853

**Published:** 2022-06-20

**Authors:** Mohd Farhan, Asim Rizvi

**Affiliations:** ^1^ Department of Basic Sciences, Preparatory Year Deanship, King Faisal University, Al-Ahsa, Saudi Arabia; ^2^ Department of Kulliyat, Faculty of Unani Medicine, Aligarh Muslim University, Aligarh, India

**Keywords:** cancer, copper, prooxidant, tumor microenvironment, polyphenol

## Abstract

Plant derived polyphenolic compounds are considered critical components of human nutrition and have shown chemotherapeutic effects against a number of malignancies. Several studies have confirmed the ability of polyphenols to induce apoptosis and regression of tumours in animal models. However, the mechanism through which polyphenols modulate their malignant cell selective anticancer effects has not been clearly established. While it is believed that the antioxidant properties of these molecules may contribute to lowering the risk of cancer induction by causing oxidative damage to DNA, it could not be held responsible for chemotherapeutic properties and apoptosis induction. It is a well known fact that cellular copper increases within the malignant cell and in serum of patients harboring malignancies. This phenomenon is independent of the cellular origin of malignancies. Based on our own observations and those of others; over the last 30 years our laboratory has shown that cellular copper reacts with plant derived polyphenolic compounds, by a Fenton like reaction, which generates reactive oxygen species and leads to genomic DNA damage. This damage then causes an apoptosis like cell death of malignant cells, while sparing normal cells. This communication reviews our work in this area and lays the basis for understanding how plant derived polyphenols can behave as prooxidants (and not antioxidants) within the microenvironment of a malignancy (elevated copper levels) and gives rationale for their preferential cytotoxicity towards malignant cells.

## Copper in Malignancies and Health

**FIGURE 1:**
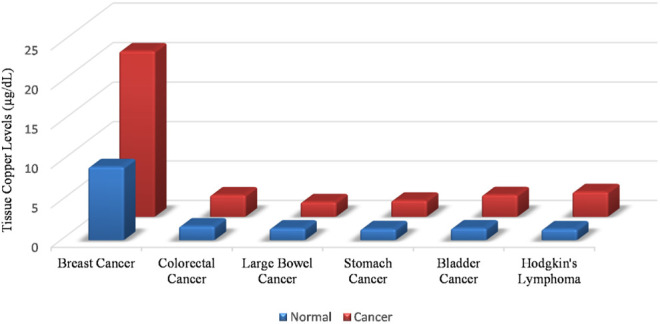
Tissue copper levels are elevated in malignancies as compared to normal tissue. This rise in cellular copper is independent of the cellular origin of the malignancy.

A meeting of the Copper Cancer Consortium held at the Cold Spring Harbour Laboratory from 1–4th March 2020 brought several aspects of the little discussed biology of copper in tumours to the forefront. “Cuproplasia” as form of copper dependent cellular proliferation was recognised, bringing a century old metabolic observation and its implications for therapy under discussion (Ge et al., 2021). Cuproplasia is a blanket term for copper dependent cell growth. It includes neoplasias and hyperplasias. Our discussion for the purpose of this review will be focused on malignant cuproplasias. Cancer cells metabolise copper differently from non-malignant cells (Rizvi et al., 2017). A peculiar feature of cancer cells is that the absolute levels of copper within malignant cells are higher as compared to non-malignant cells. This observation holds steady across malignancies of different cellular origins, across different grades of malignancies and across malignancies in different animals. In fact, it has also been observed that copper is also elevated in the serum of human beings harboring cancer. Studies from our laboratory have shown a 10 fold elevation of copper in the serum of rats harbouring diethylnitrosamine induced hepatocellular carcinoma (Rizvi et al., 2016). Figure 1 compares tissue copper levels between healthy vs. malignant individuals in various types of cancers. (Gupte and Mumper, 2009).

The dietary requirement of copper for an adult with no existing comorbidities is 0.9 mg. A normal diet is capable of fulfilling and exceeding the normal copper requirement for the body. Seed and nuts, organ meat such as liver, kidney, sweetbreads and brain are rich sources of copper along with certain sea foods and chocolate (Trumbo et al., 2001). Copper absorption largely occurs in the bolus of digested food as it passes through the small intestine. The initial cellular absorption occurs *via* CTR1 while the release of copper to the systemic circulation occurs *via* ATP7A. Within the blood stream, human serum albumin carries divalent metal ions (Chikvaidze, 1988), including copper (Suzuki et al., 1989), which is ultimately stored in the hepatocytes. The ability of hepatic storage copper is exaggerated in liver cancer where the liver may store very large amounts of copper (Fang et al., 2019). Under normal physiological conditions, copper excretion also occurs *via* the liver. ATP7B aids the cellular membrane of the hepatocyte to secrete copper, which ultimately is secreted into the digestive system *via* the bile. The biliary excretion of copper in the small intestine thus makes copper available again for uptake, if required from the small intestine (Harada et al., 2000).

Within the cell, two pools of copper exist. There is protein bound copper, which exists in micro molar concentrations and there exists bioavailable liable copper which exists in sub-femto molar concentrations (Hare et al., 2015; Ackerman et al., 2017). Of the proteinacious mediators of copper metabolism, ceruloplasmin is the major carrier of copper in blood. The copper bound to ceruloplasmin is thought of as, “exchangeable” copper. This implies that the copper bound cerruloplasmin is in a constant state of exchanging copper with other mediators and carriers of copper within the body. Other than cerruloplasmin, SL31A1 (CTR1) is responsible for copper uptake in the cell and cytoplasmic proteins such as COX11, COX17, SCO1, and SCO2 aid copper exchange between cellular proteinacious mediators. ATP7A and ATP7B have been identified as copper exporters. Thiol rich proteins such as MT1 and MT2 have high affinity sites, which can articulate multiple copper ions. Several genes are in tandem, responsible for copper homeostasis within the body (Ge et al., 2021).

Ceruloplasmin excess in plasma is often a sign of copper deficiency (Harvey et al., 2009) since it undergoes rapid degradation when it is not bound to copper. Certain inflammatory conditions also increase the levels of ceruloplasmin in the blood (Linder, 2016). In spite of being the predominant copper binding protein in the serum, ceruloplasmin is not absolutely essential for copper acquisition by tissues. Small molecular weight ligands of divalent ions such as the amino acids cysteine, histadine, methionine, aspartate, and glutamate sever as copper donors to the tissues (Gray et al., 2012; Linder, 2016). The STEAP family of enzymes, which are primarily metalloreductases help maintain copper in the Cu (I) state (Ohgami et al., 2006), which is essential for copper transport *via* CTR1.

Copper deficiency primarily manifests as Wilsons’ disease and Menke’s disease. Loss of function mutations within ATP7A, causes a loss of release of copper from the enterocytes, leading to Menke’s disease. Defects of the connective tissue, neurodegeneration, growth defects, developmental failure and hypopigmentation characterise Menke’s disease. Wilson disease occurs as a result of loss of function mutations in ATP7B, resulting in neurophysis and hepatic failure as a consequence of excess copper in the brain and the liver respectively (Członkowska et al., 2018).

Wilson disease is a risk factor for hepatocellular carcinoma in humans. Animal models of Wilsons disease have an increased susceptibility to hepatocellular carcinoma as well (Iwadate et al., 2004). This brings forth an interesting possibility that excess hepatic copper load might in some way lead to cellular transformation (Członkowska et al., 2018). On the other hand established malignancies have an abnormally high demand for copper (Blockhuys et al., 2017). By analogy, natural non diseased physiological states where cell division is accelerated, such as during pregnancy, increases the requirement of copper (Jomova and Valko, 2011). This can be attributed in part to the requirement of copper by mitochondrial cytochrome c oxidase that is required by rapidly dividing cells to produce energy. Angiogenesis, which is fundamental for any rapidly dividing cell mass, is also copper dependent. The activation of several pro-angiogenic mediators such as Interlukein 1 (IL1), Tumour necrosis factor (TNF), Fibroblast Growth Factor 2 (FGF2) and Vascular Endothelial Growth Factor (VEGF) is copper mediated. Hence, the systemic rise in copper levels during malignancies makes physiological sense.

## Fenton Chemistry Like Reactions of Copper in Malignant Cells: Evidence From Non-plant Derived Molecules

Transition metals characteristically have one electron in their outermost shell, with the notable exceptions of iron and copper. Copper in spite of its full outer shell can exchange electrons with great ease. This makes copper an ideal catalyst for redox reactions including biological redox reactions (copper catalysed aerobic reactions). Within the cell, copper exists as the oxidised (Cu^2+^) and reduced (Cu^+^) states (Uriu-Adams and Keen, 2005). The hanging redox state of cellular copper helps copper ions co-ordinate a large range of ligands such as those with carboxylate oxygen, imidazole nitrogen, cysteine thiolate, and methionine thioether groups. The presence of free copper (in the femto molar range) causes several molecules to exert a prooxidant effect with the malignant cell. The initial auto-oxidation of these molecules leads to the formation of the superoxide anion (O_2_^−^). The superoxide anion itself is highly reactive and is known to damage macromolecules (proteins, lipids and DNA). The accelerated auto-oxidation of the superoxide anion generates hydrogen peroxide within the cells, which then further decomposes to create other reactive oxygen species. It is worth noting that recent evidence suggests that the oxidative nature of a molecule is dependent on the cellular milieu. Molecules, which have been demonstrated to be known antioxidants, have shown prooxidant characteristics in certain cell types and under certain physiological conditions.

Our laboratory, and those of Prof. S.M. Hadi et al. (2000) and Prof. Imrana Naseem (Chibber et al., 2012), has been investigating this phenomenon for the last 30 years. We have shown that several standard anticancer drugs have the ability to produce free radicals in the presence of copper. Demonstrated that five Fluro-Uracil Chibber et al. (2011a) and methotrexate Chibber et al. (2011b) both produce reactive oxygen species in the presence of cellular copper and cause damage to genomic DNA leading to an apoptosis like cell death. Similarly another anticancer drug, six. Mercaptopurine (Rehman et al., 2015) has been shown to produce free radicals in the presence of copper. A large body of evidence has also shown that certain vitamins have the ability to produce free radicals in the presence of copper. Have demonstrated that ascorbic acid in the presence of copper, in the lymphocytes causes breakage of genomic DNA Ullah et al. (2011b). Further the role of cellular signalling in the anticancer effects of ascorbic acid has also been evaluated (Ullah et al., 2012).

Vitamin D, though classically associated with calcium and phosphorus metabolism and bone health, is now shown to have a potent anticancer effect. Several genomic and non-genomic mechanisms have been elucidated for the anticancer effects of Vitamin D, including reducing the number of cancer stem cells in ovarian cancer xenotransplant model (Srivastava et al., 2018).

We have shown that cholecalciferol in the presence of copper leads to the formation of hydroxyl radicals and superoxide free radical and causes genomic DNA damage in isolated human lymphocytes (Rizvi et al., 2015a). Calcitriol, the functionally active form Vitamin D has also been shown to cause DNA damage in an *ex vivo* copper loaded lymphocyte system (Rizvi et al., 2013) and in a copper overloaded mouse model (Rizvi et al., 2015b), which was developed to mimic the copper elevation in malignancies. The study was further extrapolated in the diethyl nitrosamine (DEN) induced hepatocellular carcinoma model in rats. Cooper dependent free radical generation was shown both in the animal system and in isolated malignant cells (Rizvi et al., 2016). To aid this reaction the vitamin D receptor (VDR) was hypothesised to be the chaperone molecule which brings fat soluble vitamin D in the vicinity of water soluble genomic DNA (Hasan et al., 2013).

## Antioxidant Nature of Plant Derived Polyphenolic Compounds in Cancer

The antioxidants have been shown to play a very important role in chemoprevention. Several epidemiological studies have shown that the incidence of certain cancers such as breast cancer, colon cancer, prostate cancer, and lung cancer is lower in people who have a high consumption of fruits, vegetables, and beverages. These beneficial effects are attributed to plants derived polyphenolic compounds. The polyphenol uptake can be the result of drinking green tea (catechins) (Farhan et al., 2022), consumption of large amounts of soybeans products (genistein) (Ullah et al., 2011a), berries (anthocyanins), red grapes and red wine (resveratrol), turmeric (curcumin), parsley (apigenin), and onion (quercetin) (Khan et al., 2012). The results of recent studies indicate, that these compounds serve as therapeutic molecules, thatcan aid both prevention and treatment of cancer. These naturally occurringmolecules affect a variety of signaling pathways and can exhibit a variety of biological activities, including halting cellular proliferation, promoting apoptosis and halting angiogenesis (Khan et al., 2020).

The ability of polyphenols to modulate cancer-signaling pathways is very well documented. Beneficial health effects of citrus fruit consumption has been associated with flavanones such as naringenin through the regulation of the PI3K/Akt pathway and nuclear translocation of the Nrf2 transcription factor, with the expression of HO1 (heme oxygenase 1) thereby promoting antioxidant activity (Zhang et al., 2016). Quercetin has been reported to suppress the growth of breast cancer by inhibiting glycolysis *via* the Akt mTOR pathway and activating autophagy (Jia et al., 2018). EGCG has been shown to inhibit carcinogenesis in a variety of tissues through inhibition of mitogen-activated protein kinase (MAPK), growth factor-related cell signaling, and activation of activators such as activator protein 1 (AP1) and nuclear factor B (NFκB), topoisomerase I and, matrix metalloproteinases. (Chen and Zhang, 2007). Curcumin prevents cancer by inducing phase II antioxidant enzymes through activation of the Nrf2 pathway, recovery of tumor suppressor p53, and regulation of inflammatory mediators such as iNOS and COX2 in the liver of mice with lymphoma. (Das and Vinayak, 2015).

In addition, there is increasing evidence of chemopreventive drugs being more effective in combination with phytochemicals (Jain et al., 2021). It is also worth mentioning that several combinations of plant derived polyphenols show better biological activity, which is not shown when these molecules are tested individually. (Arora et al., 2019; Jain et al., 2021).

## Role of Reactive Oxygen Species in Cancer Cell Death

ROS is associated with cancer initiation and development, and almost all forms of DNA damage (Aggarwal et al., 2019). Hydrogen peroxide (H_2_O_2_) is one of the most important inducers of apoptosis (Clément et al., 1998). Anticancer compounds have been shown to promote apoptosis *via* ROS production (Tables 1, 2) in a number of investigations (Kopustinskiene et al., 2020; Perillo et al., 2020; Khan et al., 2021; Subramaniam et al., 2021). For example, in PC-3 (prostate cancer), HepG2 (hepatic cancer), and MCF-7 (breast cancer) cells, the polyphenol resveratrol was found to cause mitochondrial H_2_O_2_ buildup by regulating antioxidant enzymes, resulting in apoptosis (Khan et al., 2013). Some evidence suggests that thymoquinone, a component of the black cumin acts as a prooxidant and induces apoptosis by generating ROS *via* various molecular pathways, such as activating Akt and causing conformational changes in the BCL-2 associated X and apoptosis regulator (Bax) protein, which results in the loss of mitochondrial membrane potential and the release of cytochrome-c, as well as activation of the caspase dependent apoptotic pathway (Hussain et al., 2011; Asaduzzaman Khan et al., 2017; Mahmoud and Abdelrazek, 2019).

**TABLE 1:** Genes responsible for copper homeostasis within the human body.

Function	Gene
Cu Metallochaperones	ATOX1 Ge et al. (2021)
	CCS Ge et al. (2021)
	COX11 Ge et al. (2021)
	COX17 Ge et al. (2021)
	SCO1 Ge et al. (2021)
	SCO2 Ge et al. (2021)
	COA6 Ge et al. (2021)
Cu Transporter	CTR1 Ge et al. (2021)
	CTR2 Ge et al. (2021)
	SLC25A3 Ge et al. (2021)
	ATP7A Ge et al. (2021)
	ATP7B Ge et al. (2021)
Cu Plasma Carrier	CP Ge et al. (2021)
Cu Storage Protein	MT1 Ge et al. (2021)
	MT2 Ge et al. (2021)

**TABLE 2:** Plant derived polyphenolic compounds act as prooxidants in the cellular microenvironment of malignant cells: Role of ROS.

Effect	Mechanism
Apoptosis	The c-Met-Nrf2-HO-1 pathway causes apoptotic cell death by increasing cell oxidation Chakraborty et al. (2019)
	Reduced ROS caused by GPx3 expression causes G2/M arrest An et al. (2018)
	ROS increases apoptosis through altering MAPK and AKT signaling, as well as DNA damage-induced p53 phosphorylation (Zhu et al. (2016)
	Increased ROS and substantial cell apoptosis due to nicotinamide nucleotide transhydrogenase knockdown Li et al. (2018a)

Most plant derived polyphenols possess both antioxidant as well as prooxidant properties (George et al., 2011; Asuzu et al., 2019; Jomová et al., 2019; Maleki Dana et al., 2022). Several studies have shown that in the presence of copper ions polyphenolic compounds can act as pro oxidants leading to cellular breakage of DNA *via* ROS formation (Said Ahmad et al., 1992; Ahsan and Hadi, 1998; Khan and Hadi, 1998; Ahmad et al., 2000; Azam et al., 2004; Ahmad et al., 2005; Farhan et al., 2015a; Arif et al., 2015). A notable example is the polyphenol resveratrol (found in grapes and berries), and its synthetic analogues (Zheng et al., 2006; Quideau et al., 2011). This can be attributed to the reduction of transition-metal ions in cells (Yoshino et al., 2004; López-Lázaro, 2007; Eghbaliferiz and Iranshahi, 2016). DNA fragmentation induced by many anticancer drugs (Tsang et al., 2003; Pelicano et al., 2004; Kim et al., 2006) is a known mechanism of chemotherapy. It has not escaped our notice, that the mechanism of DNA damage by polyphenols, in the presence of transition metals is similar to that of these anticancer drugs (Ehrenfeld et al., 1987; Santini et al., 2014).

**FIGURE 2:**
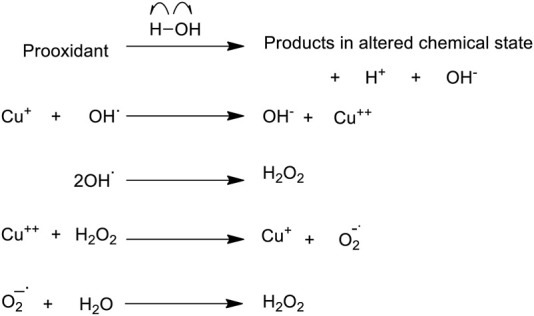
A proposed scheme demonstrating how a prooxidant may initiate formation of ROS in an aqueous environment, when copper is available.

On the basis of our own studies and of others, we proposed a new mechanism according to which plant-polyphenolic compounds mobilize intracellular copper-ions in cancer cells; leading to oxidative DNA breakage followed by cell death. The selectivity of the reaction is due to the increase in copper in cancer cells (Hadi et al., 2007) **(** Figure 2). This phenomenon is common across species (Apelgot et al., 1981; Apelgot et al., 1986) and is attributed to the role of copper in tumor etiology and growth (Finney et al., 2009; Jomova and Valko, 2011; Santini et al., 2014), including tumour revascularization and angiogenesis (Brewer, 2005). Studies in our laboratory have confirmed that both the acidic cellular environment and the binding of copper, particularly to guanine in the DNA, aid this reaction (Shamim et al., 2012).

Using intact lymphocytes and lymphocyte nuclei we have shown that plant-polyphenolic compounds mobilize copper (bound to chromatin) leading to redox-cycling of copper ions and cellular DNA breakage (Shamim et al., 2008). This finding was further substantiated by another study in which permeabilized cells were used instead of cell nuclei (Ullah et al., 2009). Permeabilized cells allow direct-interaction of polyphenols with the cell nuclei. Such a direct interaction resulted in much greater cellular DNA breakage in permeabilized cells as compared to intact cells, as anticipated. Mobilization of copper bound to chromatin and further redox cycling lead to the production of ROS and subsequent DNA breakage.

Cellular DNA degradation induced by plant-polyphenols in the presence of the Cu (I)-specific chelators neocuproine and bathocuproine was examined in intact lymphocytes as well as permeabilized lymphocytes (Ullah et al., 2009). Polyphenol-induced DNA breakage in intact lymphocytes was found to be inhibited upon incubation of lymphocytes with a cell membrane permeable copper sequestering agent (neocuproine). The membrane impermeable copper chelator (bathocuproine sulfonate) did not cause such inhibition. However, in permeabilized cells both the copper chelators were able to inhibit DNA breakage in a concentration-dependent manner. We concluded, that both neocuprione and bathocuproine sulfonate are able to cross through the permeabilized cells which allows these chelators to cross the nuclear pore-complex and facilitating a direct interaction with chromatin bound copper.

In another set of experiments, a model was designed to mimic the high copper concentrations in normal lymphocytes, by oral administration of copper to rats (Khan et al., 2011). The oral-administration of copper resulted in higher levels of copper in the serum and lymphocytes. When such lymphocytes with a copper-overload were treated with EGCG, resveratrol or genistein; an increment in cellular DNA breakage was observed. This DNA breakage could be reduced upon the addition of ROS scavengers [Super oxide dismutase (SOD) for superoxide anion, Catalase for hydrogen peroxide, which can produces hydroxyl free radicals, and thiourea for scavenging hydroxyl radicals]. Therefore, it was inferred that the involvement of nuclear copper plays an important role in cellular DNA breakage induced by plant-polyphenols and ROS play a critical role in inducing DNA breakage in this reaction.

Similar results have also been reported for other plant derived molecules such as curcumin (Ahsan and Hadi, 1998), tannic acid and its structural constituent gallic acid (Khan and Hadi, 1998), flavonoids (Said Ahmad et al., 1992), resveratrol (Ahmad et al., 2000), gallocatechins (Azam et al., 2004). We have further demonstrated that plant polyphenols can easily bind to DNA and copper ions (Rahman et al., 1990; Ahsan and Hadi, 1998). This interaction can lead to redox cycling (Hanif et al., 2008). Possibly, a ternary complex is formed when plant-polyphenols bind to DNA and chromatin bound copper. Perhaps a redox-reaction occurs between polyphenol-Cu (II) which results in the reduction of Cu^+2^ to Cu^+1^, and further re oxidation by oxygen (molecular) leads to production of ROS.

ROS is the final mediator of cellular DNA breakage (Azmi et al., 2006) and its production leads to subsequent cell death. Therefore, the redox-cycling of copper-ions is an essential physiological paradigm for copper-mediated cellular DNA damage induced by plant polyphenols.

## Plant Derived Polyphenolic Compounds Cause Cellular DNA Breakage in the Presence of Copper Ions *in-vivo*

In order to confirm our hypothesis *in-vivo*, hepatocellular carcinoma (HCC) was induced chemically in rats by diethyl nitrosamine (DEN) (Farhan et al., 2015b). A progressive increase in intracellular copper was observed after DEN treatment. ROS mediated DNA damage was also seen in these HCC cells, upon exposure to polyphenols. The cell membrane-permeable copper-chelator (neocuproine) inhibited the EGCG-mediated cellular DNA breakage in these cells. The membrane-impermeable copper chelator (bathocuproine) was found to be ineffective. Similarly, specific iron chelator (desferoxamine mesylate) and zinc chelator (histidine) were also found to be ineffective in inhibiting EGCG-mediated cellular DNA breakage.

## Role of Copper Transporters

Recently, we showed that culturing normal breast epithelial cells (MCF10A) in a medium enriched with copper sensitizes these cells to polyphenol mediated growth inhibition. This sensitization also leads to an increased expression of both CTR1 and ATP7A (membrane bound copper transporters) (Farhan et al., 2022). The over-expression led to further accumulation of more copper in such cells (Brewer, 2005; Farhan et al., 2022). siRNA mediated silencing of the copper-transporter CTR1 led to a reduction in the sensitivity of these cells to EGCG mediated cell death. (Farhan et al., 2022).

## Using Elevated Copper in Cancer Therapy

The idea that increased levels of cellular copper can be used as a target for cancer therapy is not new. Several efforts have been directed towards developing copper chelation therapies, which have yielded some interesting results. Though a detailed discussion of copper chelation therapy in cancer is beyond the scope of this review, however some interesting developments in the this field include, the discovery that patients refractory to platinum drug based chemotherapy can have copper levels in the serum which go up to 160%, as compared to patients who responded to platinum drug based chemotherapy (Majumder et al., 2009). This brings forth the paradigm that could increase in copper levels be a cause/effect of drug resistance in cancer.

It has now been shown that copper transport proteins may play an important role in the influx, efflux and bioaccumulation of platinum based chemotherapeutic drugs. CTR1 has been shown help transport of platinum based drugs into the malignant cell, and therefore its cellular expression has been linked drug sensitivity (Kuo et al., 2012). Similarly, ATP7A and ATP7B have been associated with efflux of platinum-based chemotherapeutic drugs. This leads to reduced cellular accumulation of the chemotherapeutic molecule and hence, higher cellular expression of ATP7A and ATP7B has often been correlated to resistance to platinum based drugs (Li et al., 2018b). Yet another level of complexity to this regulation is added by the fact that the expression and activity of all three copper transporters in question, that is CTR1, ATP7A, and ATP7B are themselves modulated by intracellular copper levels. Therefore when copper chelation therapy reduces the copper levels within the cell, it in turn, increases CTR1 levels and reduce ATP7A levels, which leads to cellular accumulation of the drug and a consequent increase in the efficacy of the chemotherapy is observed (Fu et al., 2012). Several clinical trials have been performed to evaluate copper chelation therapy and its role in platinum based drug resistance for cancer (Fu et al., 2012; Huang et al., 2019). The involvement of copper transporters, the dependence of resistance on copper homeostasis within the tumour and at the cellular level, and the lack of definitive clinical progress in copper chelation therapy for cancer calls for utilizing increased cellular levels of copper within the malignancy for alternative pharmacological interventions. Polyphenols in this regard, can serve as lead molecules for development of safe chemotherapeutic molecules, which utilize cellular copper to distinguish between malignant and non-malignant cells for targeted chemotherapy.

## The Structure-Activity Relationship in Polyphenols

The structure–activity relationship between genistein and biochanin A, two related isoflavones, was investigated. Genistein was discovered to be a more efficient prooxidant and antioxidant than biochanin A. The number of hydroxyl substitutions in a flavonoid’s backbone structure has been shown to affect both its prooxidant and antioxidant properties (Platzer et al., 2022). Modification of the hydroxyl groups has also been found to affect the biological activity of phenolics. The methylation of the 3-hydroxyl group in protocatechuic acid to create vanillic acid, for example, resulted in a substantial reduction in radical scavenging capacity (Simić et al., 2007). The synthesis of 3,40-dimethylether or 3,40,7-trimethylether from kaempherol (3,40,5,7 tetrahydroxyflavone) was also observed to inactivate the prooxidant activity.

The number and positions of hydroxyl groups in the flavonoid skeleton has also been found to play a role in the degree of cellular DNA damage that the flavonoid causes in the cell. The flavonoid structure consists of three group pairs that can create copper chelates (Cao et al., 1997; Arif et al., 2015). The remaining hydroxyl groups in the skeleton, in addition to the three copper complexing locations, may also contribute to DNA breakage by forming a quinone-like molecule inside the cell. This almost certainly contributes to the production of H_2_O_2_ in both the cytoplasm and the nucleus. The findings also point to the role of ortho-dihydroxy groups in the B-ring (such as 3′–4′ or 4′–5′) in the actions of myricetin, fisetin, and quercetin on cellular DNA degradation and generation of H_2_O_2_ (Cao et al., 1997; Arif et al., 2015; Arif et al., 2018). This is also supported by the fact that galangin, which lacks these hydroxyls, is the least effective in breaking down cellular DNA.

The orientation and number of hydroxyl groups in the catechin skeleton has been found to be crucial in influencing the degree of cellular DNA breakage. Hydroxyls that are orthogonal to each other may also have a role in Cu^+2^ chelation and reduction to Cu^+1^ (Farhan et al., 2015a). Furthermore, the number of galloyl moieties appears to be important, as demonstrated by the greater efficacy of EGCG as compared to EGC, in inducing DNA breakage. It is understood that the hydroxyl radical must be formed in close proximity to cellular DNA in order to cleave DNA. This is due to the hydroxyl radical’s narrow diffusion radius and thus its strong reactivity (Pryor, 1988). The differential in cellular DNA breakage caused by (+)-catechin and (−)-epicatechin is intriguing because these two molecules are stereo isomers with the hydroxyl group at position three in the C ring oriented differently. It appears that the order of fluorescence increase of the four catechins on binding to DNA follows the progressive pattern of C, EC, EGC, EGCG, indicating that their placement on chromatin with regards to copper ions is also important (Farhan et al., 2015a; Farhan et al., 2016).

## Designing Polyphenol Based Drugs Which Utilize Increased Levels of Copper Within Malignant Cells

The concept of using plant derived polyphenolic compounds for cancer chemotherapy has been under discussion for some time now. The research focus has largely remained on naturally occurring dietary polyphenols. However, the following aspects need to be addressed before viable chemotherapeutic molecules derived from plant-derived polyphenolics can be brought to the clinic.

## Bioavailability

While most coloured fruits and vegetables have a sizable polyphenolic fraction, the bioavailability of these molecules remains low in the system. In this regard, two major considerations need attention. Firstly, dietary polyphenols tend to undergo hydrolysis by enzymes in the intestine before absorption can take place. A large proportion of the total polyphenolic molecules contain several hydroxyl groups, which subsequently undergo sulfatation, glucuronidation and methylation. It is important to point out at this juncture, that hydroxyl groups and their relative positions within the molecule are shown to have fundamental importance in the copper based ROS mediated mechanism that we have proposed (Pryor, 1988; Cao et al., 1997; Farhan et al., 2015a; Farhan et al., 2016; Arif et al., 2018; Farhan et al., 2022). Secondly, it is estimated that the intestinal absorption of polyphenols as such tends to range between 5 and 10% of the total polyphenolic content. The remaining polyphenolic fraction, even if available in the intestine as such, tends to accumulate in the large intestine and undergoes excretion in the feces. Even during the process of absorption, polyphenols tend to conjugate with other small molecules in the small intestine and are later acted upon by the liver (Chen et al., 2018). Clearly dietary polyphenols will thus be insufficient to bring about the desired pharmaceutical effect. For example, despite the promising results recently published by our laboratory of epigallocatechin-3-gallate (EGCG) (Farhan et al., 2022), the oral bioavailability of EGCG remains exceedingly low (Mereles and Hunstein, 2011). The reasons attributed to its low bioavailability include, transporter protein linked efflux in the intestinal cells and its poor permeability across the bilipid layer (Kanwar et al., 2012).

## Retention Time

Dietary polyphenolics have variable retention time, which is often structure dependent. However, even during maximum retention, the retention time is insufficient to achieve pharmaceutically relevant concentrations of plant-derived polyphenols within the body. Acute treatment shows little and/or insignificant circulatory levels, while chronic treatment with polyphenols often results in observable changes in the circulatory levels of polyphenols (Curti et al., 2019). However even at these levels most of the polyphenol usually exists conjugated with other small molecules. Studies have been undertaken for increasing the retention of resveratrol. It was shown that chitosan loaded emulsions adhered better to mammalian intestinal mucosa, and thereby increasing uptake (Jo et al., 2021). However, the pharmacological capabilities of such conjugates, in our context have not been understood in great detail.

## Tumour Targeting

Even if pharmacologically relevant levels of plant derived polyphenolics and their active conjugates are achieved in blood, there exists a need to target these molecules to the tumour site to achieve the maximum therapeutic effect. Tumour targeting has been discussed in great detail in other contexts (Iyer et al., 2006). Metabolically linked homing of polyphenol and/or its pharmacological relevant conjugate to the tumour is another strategy that can be developed for this purpose. Tumour targeting of poorly soluble polyphenols with a high potential for anticancer activity has been achieved, with some success using nano formulations (Bonferoni et al., 2017). Notable examples include nano formulations of quercetin, curcumin and resveratrol (Bonferoni et al., 2017). Alternatively, over loading strategies within safe limits can be used to effectively deliver the desired concentration of plant-derived polyphenolics to the tumour.

## Structural Considerations and Alternative Anticancer Mechanisms

For the copper mediated Fenton like reaction, which we have been describing for the last several years, the structure of the polyphenol, most notably the position of the hydroxyl groups attached to the aromatic core is indispensable. While developing candidate lead molecules, which can be, developed as full-fledged chemotherapeutic agents, the pharmacologically active structural motifs which aid the generation of ROS by the mechanism described by us need to be conserved. It may also be noted that plant derived polyphenolics exert their anticancer effect by several known and experimentally verified mechanisms other than the mechanism described by us. To this end, it will be interesting to develop lead molecules which utilize more than one mechanism to exert their anticancer effect. Ideally, such a lead molecule should have several mechanisms for their anticancer effects, so that refractory tumours and tumours cells with more than one escape mechanism can also be targeted. Several polyphenols have shown that they utilize more than one pathway to mediate their anticancer effect. Green tea polyphenol, EGCG has been shown to influence cell cycle arrest, inhibit Necrosis Factor κ B (NFκB) pathway, inhibit MAP Kinase pathway and Activation Protein 1, besides inhibiting Epidermal Growth Factor Receptor mediated signaling (Khan et al., 2006). Besides these pathways, Insulin like Growth Factor 1 mediated signalling, overexpression of cyclooxygenase 1 (COX1) and alteration of Proteosome activity is also mediated by EGCG exposure. Angiogenesis, migration and metastasis are influenced by EGCG, by altering the vascular endothelial growth factor signaling, the activity of matrix metalloproteases and the activity of urokinase plasminogen activator (Khan et al., 2006). Structure based drug designing to modulate the pharmacological activity of plant derived polyphenols has been an area of interest for our laboratory. Using an *in silico* screen we have been able to identify plant derived molecules which can modulate the oxidative stress response in malignant cells by altering the structure of transcription factors that mediate the oxidative stress cascade (Rizvi et al., 2021). Similar studies are in progress in our laboratory, where we aim to develop polyphenol based molecules, which can mediate anticancer effects by more than one mechanism.

## Conclusion

The cellular microenvironment of the malignant cell, (that is, the increased level of copper and the increased acidity that makes guanine bound copper available for reacting) allows polyphenols to generate ROS in the vicinity of DNA. This leads to genomic DNA damage and apoptosis like cell death of the malignant cell, while sparing normal cells. Our work in this area for the last 30 years, has led us to understand this phenomenon very well. The major milestones have been summarized in Table 3. This understanding of the prooxidant behaviour of polyphenols not only provides a novel paradigm to understand the mechanism behind the anticancer activity of plant polyphenols with drastically different chemical structures, but also provides a strong basis for utilizing the high-copper redox status of malignant cells as a molecular target for designing more potent cancer therapeutic strategies. The caveat however, is that the molecular structure of plant derived polyphenolic compounds, their metabolism, their bioavailability and retention time, prove to be a hindrance in the utilization of these molecules in the clinic. In this context, it has not escaped our attention that plant derived polyphenolic compounds are usually discussed in context of dietary polyphenols and their therapeutic effects. In addition to their benefits in the concentrations available through the diet, these molecules can serve as lead molecules, which can further be developed in such a manner so that their efficacy for treatment can be enhanced, and they can eventually be utilized in the clinic for chemotherapy.

**TABLE 3:** Molecular findings to support the copper mediated prooxidant anticancer activity of plant polyphenols.

S. No.	Molecular finding	References
I.	A reaction between polyphenols, Cu (II) and DNA results in DNA breakage *in-vitro*	Ahmad et al. (2000)
		Ahsan and Hadi (1998); Khan and Hadi (1998); Azam et al. (2004)
		Said Ahmad et al. (1992)
II.	Polyphenol-Cu (II) system leads to DNA breakage in a cellular system	Azmi et al. (2005)
III.	Polyphenols can mobilize endogenous copper ions from cells resulting in cellular DNA degradation	Azmi et al. (2006)
		Bhat et al. (2007)
IV.	Nuclear copper is mobilized in the polyphenol induced oxidative cellular DNA degradation	Shamim et al. (2008)
		Ullah et al. (2009)
V.	Oral administration of copper to rats results in enhanced prooxidant cellular DNA breakage by polyphenols	Khan et al. (2011)
VI.	Diethylnitrosamine induced hepatocellular carcinoma in rats leads to increased prooxidant cellular DNA breakage by polyphenol (EGCG)	Farhan et al. (2015b)
VII.	Polyphenols induce growth inhibition and apoptosis in malignant cells through copper redox-cycling and ROS generation	Ullah et al. (2011a)
		Khan et al. (2014)
		Zubair et al. (2016)
VIII.	Supplementation with copper sensitizes normal breast epithelial cells to antiproliferative activity of polyphenols	Farhan et al. (2016)
		Khan et al. (2014)
IX.	Silencing of copper transporter CTR1 desensitize normal breast epithelial cells in copper enriched medium to antiproliferative activity of polyphenol (EGCG)	Farhan et al. (2022)
